# Tight glycemic control in critically ill pediatric patients: a systematic review and meta-analysis

**DOI:** 10.1186/s13054-018-1976-2

**Published:** 2018-03-04

**Authors:** Lvlin Chen, Tiangui Li, Fang Fang, Yu Zhang, Andrew Faramand

**Affiliations:** 1 Department of Critical Care Medicine, Affiliated Hospital of Chengdu University, No.82, North Section 2, 2nd Ring Road, Jinniu District, Chengdu, Sichuan 610081 China; 2 Longquanyi Hospital, Chengdu, Sichuan China; 3 West China Hospital, Sichuan University, Chengdu, Sichuan China; 4 University of Pittsburgh Medical Center, Pittsburgh, PA USA

**Keywords:** Hyperglycemia, Randomized controlled trial, Children, Tight glycemic control, Mortality

## Abstract

**Background:**

Hyperglycemia is prevalent in patients in the pediatric intensive care unit. The purpose of this study was to describe the benefits and risks of tight glucose control (TGC) in critically ill children.

**Methods:**

A systemic review and meta-analysis of the literature was carried out on randomized controlled trials of TGC in critically ill children admitted to the pediatric intensive care unit. The databases searched were Medline, Embase, and CENTRAL databases until May 1, 2017. Paired reviewers independently screened citations, assessed risk of bias of included studies, and extracted data. A random-effects model was used to report all outcomes. The Grading of Recommendations Assessment, Development and Evaluation system was used to quantify absolute effects and quality of evidence. The primary outcome was hospital mortality**.** The secondary outcomes were hypoglycemia (any, severe), sepsis, new need for dialysis, and seizures.

**Results:**

A total of 4030 patients were included from six studies. All six studies were rated as at low risk of bias. Our meta-analysis showed that TGC did not result in a decrease in risk of hospital mortality (odds ratio (OR), 0.95; 95% confidence interval (CI), 0.62–1.45; *I*^2^ = 40%; moderate quality), sepsis (OR, 0.82; 95% CI, 0.63–1.08), or seizures (OR, 0.98; 95% CI, 0.59–1.63). TGC was associated with a decrease in new need for dialysis (OR, 0.63; 95% CI, 0.45–0.86). However, TGC was associated with a significant increase in any hypoglycemia (OR, 4.39; 95% CI, 2.39–8.06) and severe hypoglycemia (OR, 4.11; 95% CI, 2.67–6.32).

**Conclusions:**

Among critically ill children with hyperglycemia, TGC does not result in a decrease in hospital mortality, but appears to reduce a new need for dialysis. However, TGC is associated with higher incidence of hypoglycemia.

**Systematic review registration:**

PROSPERO registration number CRD42017074039.

**Electronic supplementary material:**

The online version of this article (10.1186/s13054-018-1976-2) contains supplementary material, which is available to authorized users.

## Background

Hyperglycemia is prevalent in patients in the pediatric intensive care unit (PICU), with more than 80% having a blood glucose concentration greater than 110 mg/dl, more than 60% a concentration greater than 150 mg/dl, and more than 30% a concentration exceeding 200 mg/dl [1–4]. The extent of hyperglycemia is associated with adverse outcomes, including organ failure, length of stay in the PICU, and death [1, 5–9]. Consequently, the practice of tight glucose control (TGC) with insulin treatment in critically ill children has emerged as a plausible strategy to improve outcomes. To achieve such ambitious goals in clinical practice, however, there are significant challenges in increased risk of hypoglycemia, additional personnel training, efficient utilization of medical resources, and radical revamping of glycemic management protocols [10]. Furthermore, insulin treatment for critically ill patients only works when the normal healthy fasting ranges for blood glucose concentrations are achieved, and these are lower in children than in adults [11].

Several investigations have examined the benefits and risks of using TGC in critically ill children. In 2009, Vlasselaers et al. [12] published a single-center, randomized controlled trial (RCT) of critically ill children showing that TGC of 80–110 mg/dl reduced hospital mortality by half, and reduced the infection rate and length of stay, but also presented extremely high rates of severe hypoglycemia. However, subsequent multicenter large RCTs of TGC have failed to replicate this mortality benefit [13, 14]. Furthermore, a recent trial, the Heart and Lung Failure—Pediatric Insulin Titration (HALF-PINT) trial, was stopped early because the data indicated a low likelihood of benefit and evidence for the possibility of harm [15].

A meta-analysis [16] has been published on this subject. However, results from two RCTs [15, 17] were not included in the study. Moreover, the meta-analysis failed to conducted subgroup analyses on critical variables or a formal evaluation of the quality of evidence (using the Grading of Recommendations Assessment, Development and Evaluation (GRADE)). Thus, a comprehensive overview of all RCTs involving critically ill children has never been performed, and the optimal glucose goal remains largely unknown.

Consequently, the considerable controversy of RCTs and the limitations of the prior meta-analysis prompted us to perform an updated systematic review and meta-analysis examining the risks and benefits of TGC as compared with usual care in critically ill children. Moreover, we conducted subgroup analyses on three variables that have been debated in the controversy over TGI: glucose goal (< 110 mg/dl or 110–140 mg/dl), patient setting (cardiac surgery or not cardiac surgery), and continuous glucose monitoring.

## Methods

### Protocol and guidance

The study protocol was prepared following PRISMA-P guidelines [18] and was registered at PROSPERO (CRD42017074039). The methods of the systematic review and meta-analysis followed PRISMA guidelines [19]. Reporting of statistical data in the study followed SAMPL guidelines [20].

### Study selection

#### Inclusion criteria

We included RCTs that met each of the following criteria: the setting was a PICU, and the patient was child (age < 16 years); the intervention group received TGC (glucose goal < 140 mg/dl obtained using insulin treatment during part or all of the PICU stay); the comparison group received usual care (method of insulin administration and glucose goal could vary between trials); and the primary or secondary outcomes included hospital mortality, hypoglycemia (any, severe), new need for dialysis, sepsis, or seizures.

#### Exclusion criteria

Trials were excluded if the intervention was conducted primarily during the intraoperative period rather than during the PICU stay, or if we were unable to obtain adequate details of the study methodology or results from the article or study investigators.

#### Missing data

We contacted the investigators of all unpublished RCTs as well as any published RCTs in which data were missing to confirm eligibility and obtain additional study details.

#### Duplicate publications

If separate articles from the same RCT were published, the article with the most updated data was selected. In the case of duplicate publications, only one publication was included.

#### Information sources and search strategy

Medline, Embase, and the Cochrane Library at the CENTRAL Register of Controlled Trials were systematically searched. Gray literature was searched through appropriate databases (British Library Thesis Service, Database of Abstracts of Reviews of Effects, OpenGrey). We also consulted databases of clinical trial registries (ClinicalTrials.gov, World Health Organization International Clinical Trials Registry Platform, European Union Clinical Trials Register, ISRCTN Registry). The last electronic search was on May 1, 2017. We also hand searched the references to the retrieved articles and meta-analyses.

For the search strategy, we used a combination of keywords and MeSH terms for “child” AND “insulin”, using the sensitive search filters for therapeutic interventions (Additional file 1: Supplemental Digital Content).

#### Study selection

Two reviewers (YZ and LC) independently screened the titles and abstracts of retrieved reports for potential eligibility. They then screened the full text of potentially relevant trials. Disagreements were resolved by discussion and consensus or by consulting a third reviewer (TL).

#### Data collection process

Following removal of duplicate articles, two reviewers (YZ and LC) independently extracted data from the included RCTs using a standardized electronic form. Disagreements between the two reviewers were resolved by discussion and consensus or by consulting a third reviewer (TL). Another reviewer (FF) double-checked the extracted data.

### Outcomes and prioritization

The primary outcome was hospital mortality because we considered a reduction in hospital mortality to be the most important potential benefit of TGC. Hospital mortality was defined as death occurring during the hospital stay or within 30 days following admission. In cases in which both in-hospital and 30-day outcomes were reported, the former was used for analysis.

The secondary outcomes were hypoglycemia (any, severe), sepsis, new need for dialysis, and seizures. We defined severe hypoglycemia as a blood glucose level below 40 mg/dl and any hypoglycemia as a blood glucose level below 60 mg/dl. We defined sepsis to encompass the terms septicemia, bacteremia, or a description of positive blood cultures; a general description of infection did not qualify.

### Subgroup and sensitivity analyses

We performed subgroup analyses based on three variables prespecified clinically relevant to analysis outcomes: glucose goal in the tight control group, cardiac surgery, and continuous glucose monitoring. Subgroup analyses were performed only if there were at least two RCTs in each subgroup or a trial’s report permitted a comparison within the trial.

Differing opinions exist on the optimal level of TGC. The 2018 recommendations from the American Diabetes Association recommend targeting blood glucose levels of 140–180 mg/dl in critically ill patients [21–23]. We stratified studies by glucose goal in the TGC group into two categories: very tight control (upper limit of glucose goal < 110 mg/dl); and moderately tight control (upper limit of glucose goal 110–140 mg/dl).

Because of the concern that the pathophysiological effect of hyperglycemia may differ between patients with and without cardiac surgery, we stratified trials by PICU setting into two categories: cardiac surgery and not cardiac surgery. For trials involving mixed populations but not presenting separate data for patients with cardiac surgery, we included the pooled results in the cardiac surgery subgroup only if ≥ 50% of patients underwent cardiac surgery.

Continuous glucose monitoring has been shown to be safe and effective in children and adults, and may assist in the safer provision of tight glycemic control, with less hypoglycemia [24]. Thus, we stratified trials by whether they used continuous glucose monitoring to control blood glucose.

We conducted sensitivity analyses to examine the impact of using alternative effect measures (odds ratio vs relative risk), pooling methods (Peto vs Mantel–Haenszel (M–H) or inverse variance), statistical models (fixed vs random effects), and removing one study at a time.

### Risk of bias and quality of evidence

Two reviewers (YZ and LC) independently assessed risk of bias (low risk of bias, high risk of bias, or unclear risk of bias) using the Cochrane risk of bias instrument, which deals with random sequence generation and allocation concealment (selection bias), blinding of study participants and personnel (performance bias), blinding of outcome assessment (detection bias), incomplete outcome data (attrition bias), selective reporting (reporting bias), and other bias. They resolved any disagreements by discussion and consensus or by consulting a third reviewer (TL). We judged trials with more than two high-risk components as having a moderate risk of bias, and trials with more than four high-risk components as having a high risk of bias. We used the GRADE approach to rate the quality of evidence and generate absolute estimates of effect for the outcomes [25].

### Data synthesis

Computations were performed with RevMan 5.3.3 software (freeware available from The Cochrane Collaboration). We used the M–H method as the primary analysis to estimate the odds ratio (OR) and 95% confidence intervals (CIs). Two-tailed *P* < 0.05 was considered a criterion for statistical significance. We report the results of the random-effects model for all outcomes. We assessed heterogeneity with the Cochran *Q* test and the *I*^2^ test, with *I*^2^ values exceeding 25%, 50%, and 75% representing low, moderate, and high heterogeneity, respectively [26]. If an analysis included 10 or more RCTs, we planned to use a funnel plot to explore the possibility of published bias.

## Results

### Search results and characteristics of included studies

**Fig. 1:**
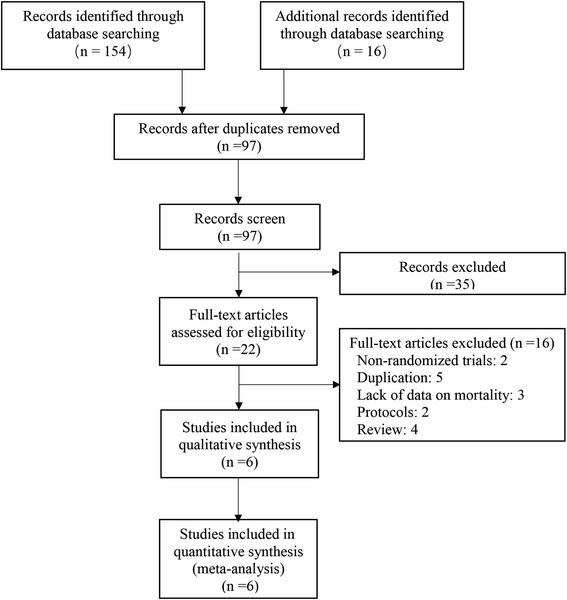
Study selection for inclusion in meta-analysis

The literature search yielded 154 articles, of which 22 were reviewed in full text (Fig. 1). Of these articles, six RCTs [12–15, 17, 27] met the inclusion criteria. The six included trials randomized 4030 patients (1980 to tight glycemic control and 2050 to receiving control) (Table 1). Trials were conducted in a diverse array of countries, half of them at a single center. Study sizes ranged widely (88–1369 patients), with two trials enrolling fewer than 300 patients and four trials enrolling more than 700 patients. The study participants encompass a broad distribution of critically ill children (Vlasselaers et al. [12], mixed with 75% cardiac surgery; Macrae et al. [13], mixed with 60% cardiac surgery; Jeschke et al. [27], severe burns; Agus et al. [14], cardiac surgery; Agus et al. [15], noncardiac surgery; Alsweiler et al. [17], preterm). TGI, as well as mean achieved glucose levels, varied between trials in both the tight control and usual care groups (Additional file 1: Supplemental Digital Content Table S1).

**Table 1:** Characteristics of studies comparing tight and usual glucose control

Author	Year	Size (*n*)	Centers (*n*)	Country	Setting	Age, median (IQR)	
						Tight control	Usual control
Vlasselaers et al. [12]	2009	700	1	Belgium	Mixed with 75% cardiac surgery	1.4 (0.3–5.5)	1.3 (0.3–4.6)
Jeschke et al. [27]	2010	239	1	USA	Burns	7.7 (5.2)^a^	10.8 (5.4)^a^
Agus et al. [14]	2012	980	2	USA	Cardiac surgery	0.4 (0.2–0.8)	0.4 (0.2–0.9)
Alsweiler et al. [17]	2012	88	1	New Zealand	Preterm	Preterm babies	
Macrae et al. [13]	2014	1369	13	UK	Mixed with 60% cardiac surgery	0.5 (0.1–2.7)	
Agus et al. [15]	2017	713	35	USA	Mixed without cardiac surgery	5.5 (1.4–12.5)	6.7 (1.7–12.8)

*IQR* interquartile range

^a^Mean (standard deviation)

### Risk of bias and quality of evidence

**Fig. 2:**
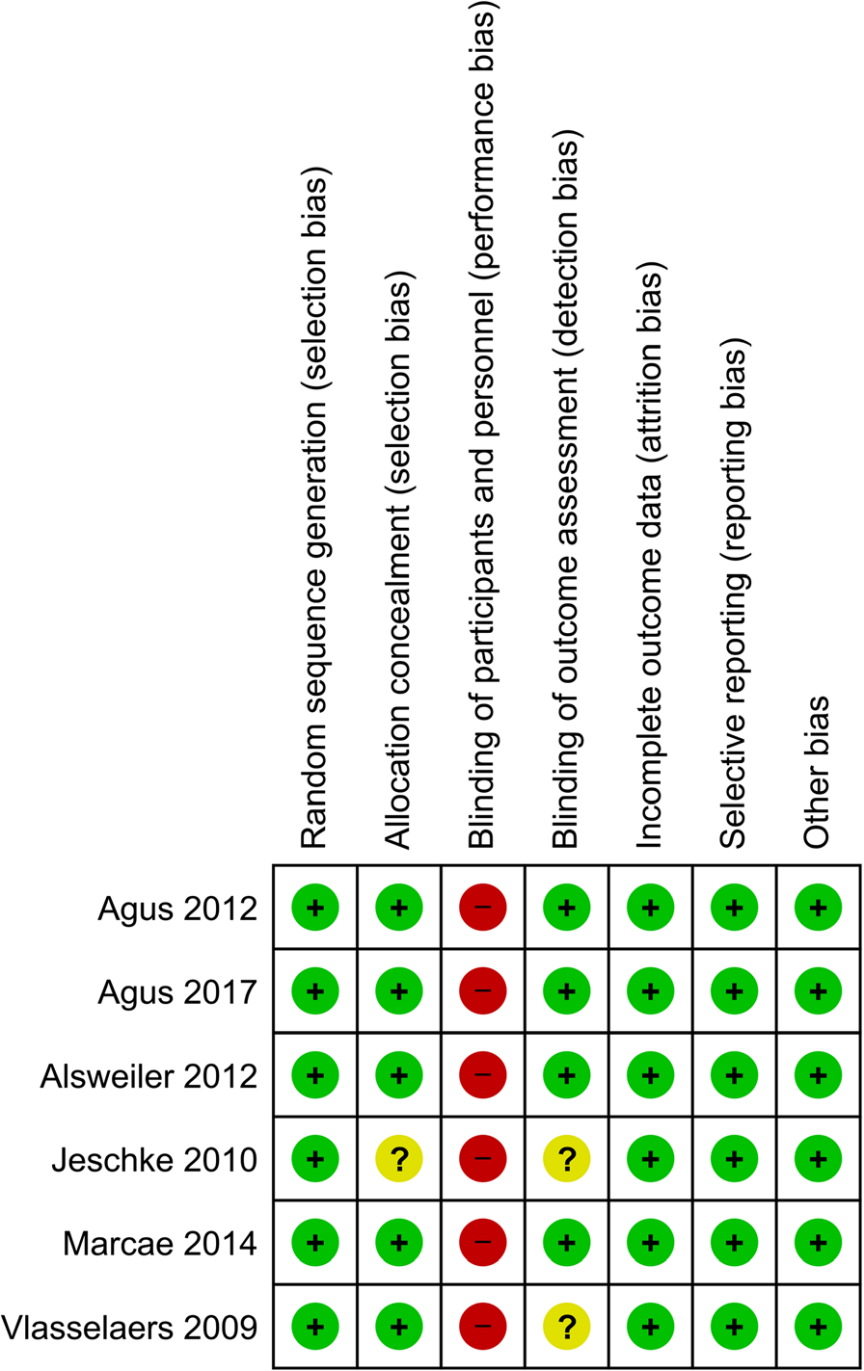
Risk of bias summary. Agus 2010 [14], Agus 2017 [15], Alsweiler 2012 [17], Jeschke 2010 [27], Macrae 2014 [13], Vlasselaers 2009 [12]

Treating clinicians were not blinded to treatment allocation in any of the trials. Most investigators were, however, blinded to treatment and outcomes. Study quality appraisal indicated that studies were of variable quality (Fig. 2) and that all six trials had a low risk of bias. Table 2 presents GRADE summary findings for all outcomes.

**Table 2:** GRADE evidence profile of outcomes, tight glucose control vs usual glucose control

Outcome	Number of children (studies)	Effect		Quality^a^	Importance
		Relative effect, OR (95% CI)	Absolute risk (95% CI)		
Hospital mortality	4021 (6 studies)	0.95 (0.62–1.45)	3 fewer per 1000 (from 20 fewer to 23 more)	⊕ ⊕ ⊕⊝ moderate^b^	Critical
Severe hypoglycemia (glucose < 40 ml/dl)	3835 (5 studies)	4.11 (2.67–6.32)	42 more per 1000 (from 23 more to 69 more)^b^	⊕ ⊕ ⊕ ⊕ high	Critical
Any hypoglycemia (glucose < 60 mg/dl)	3747 (4 studies)	4.57 (2.24–9.33)	157 more per 1000 (from 61 more to 299 more)	⊕ ⊕ ⊕ ⊕ high^b^	Critical
Dialysis	3049 (3 studies)	0.63 (0.45–0.86)	24 fewer per 1000 (from 9 fewer to 36 fewer)	⊕ ⊕ ⊕ ⊕ high^b^	Important
Seizures	3047 (3 studies)	0.98 (0.59–1.63)	0 fewer per 1000 (from 8 fewer to 12 more)	⊕ ⊕ ⊝⊝ low^b,c^	Important
Sepsis	4021 (6 studies)	0.83 (0.63–1.08)	20 fewer per 1000 (from 45 fewer to 9 more)	⊕ ⊕ ⊕ ⊕ high	Important

*GRADE* Grading of Recommendations Assessment, Development and Evaluation, *OR* odds ratio, *CI* confidence interval

^a^High quality, further research is very unlikely to change our confidence in the estimate of effect; moderate quality, further research is likely to have an important impact on our confidence in the estimate of effect and may change the estimate; low quality, further research is very likely to have an important impact on our confidence in the estimate of effect and is likely to change the estimate; very low quality, we are very uncertain about the estimate

^b^Wide confidence

^c^High heterogeneity

### Primary outcome: hospital mortality

**Fig. 3:**
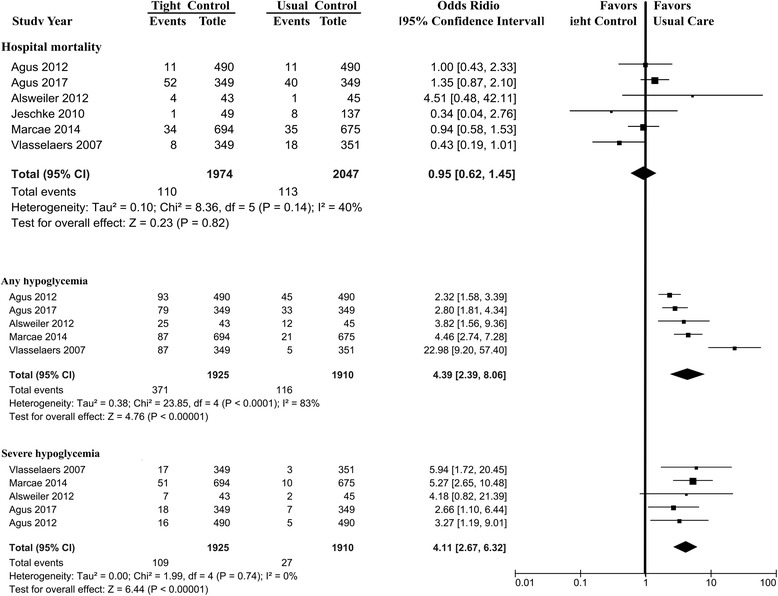
Association of tight glucose control vs usual glucose control with hospital mortality, any hypoglycemia and severe hypoglycemia. CI confidence interval, Agus 2010 [14], Agus 2017 [15], Alsweiler 2012 [17], Jeschke 2010 [27], Macrae 2014 [13], Vlasselaers 2009 [12]

Hospital mortality was reported in six trials. These trials reported 223 deaths (TGC, 110/1974 (5.6%); UGC, 113/2047 (5.5%)). Our meta-analysis showed no significant difference in mortality between tight control vs usual control (OR, 0.95; 95% CI, 0.62–1.45; *P* = 0.82; *I*^2^ = 40%; Fig. 3). Tests for heterogeneity identified the trial by Vlasselaers et al. [12] as having outlying results, which appeared to be explained by the lowest glucose target in the six trials. Exclusion of the outlying trial resolved this heterogeneity (*I*^2^ = 0%, *P* = 0.40), but did not significantly change the findings (OR, 1.13; 95% CI, 0.84–1.52).

Sensitivity analyses using an alternative statistical method, effect measure, analysis model, and after removing one study at a time showed similar results for hospital mortality.

### Secondary outcomes: hypoglycemia, sepsis, new need for dialysis, seizures

Hypoglycemia was reported in five published trials. TGC was associated with an increased risk of severe hypoglycemia (OR, 4.11; 95% CI, 2.67–6.32; *I*^2^ = 0%; 42 more per 1000 patients; high quality) and any hypoglycemia (OR, 4.39; 95% CI, 2.39–8.06; *I*^2^ = 83%; 157 more per 1000 patients; high quality) (Table 2 and Fig. 3).

**Fig. 4:**
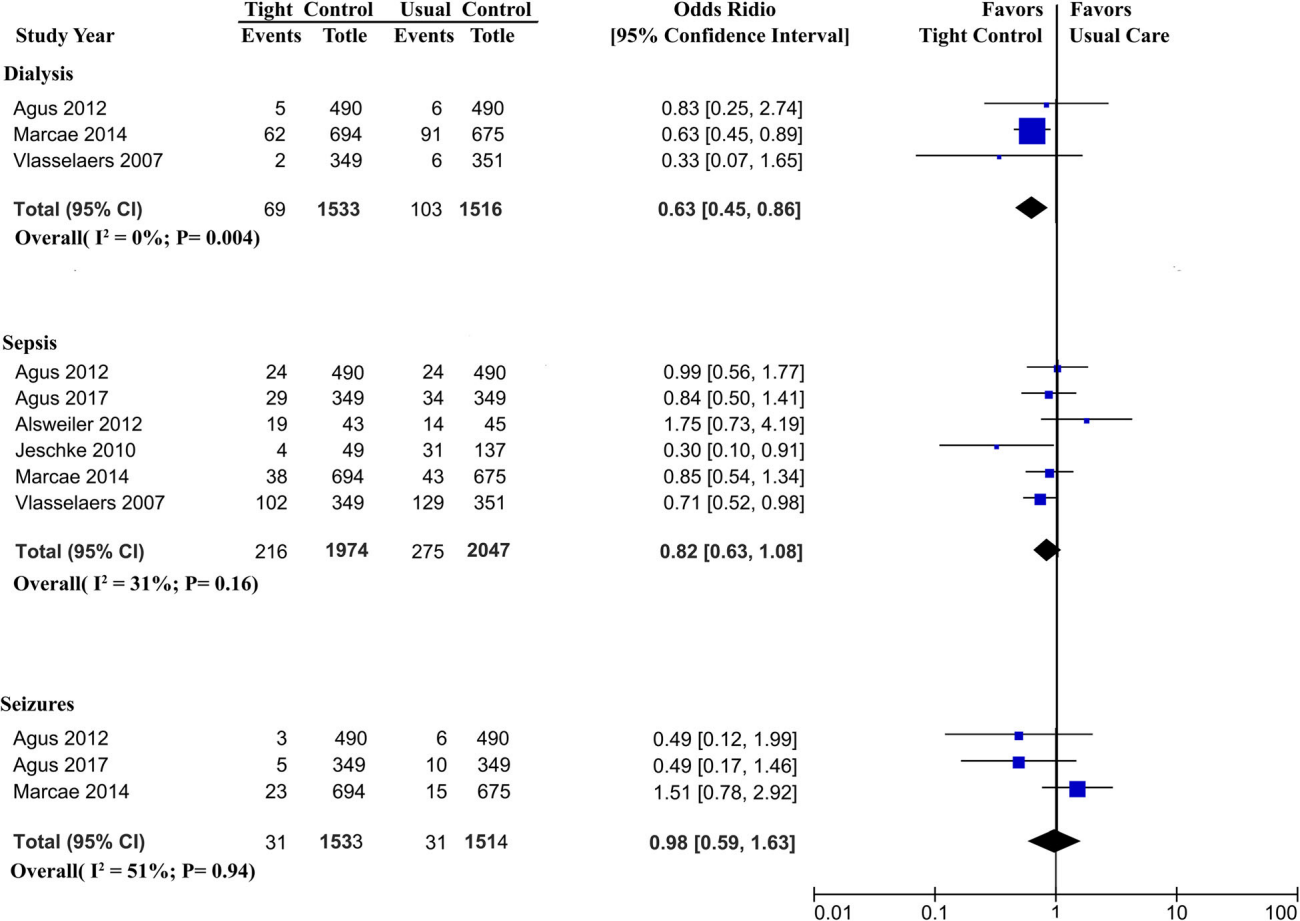
Association of tight glucose control vs usual glucose control with sepsis, new need for dialysis, seizures. CI confidence interval, Agus 2010 [14], Agus 2017 [15], Alsweiler 2012 [17], Jeschke 2010 [27], Macrae 2014 [13], Vlasselaers 2009 [12]

New need for dialysis was reported in three trials, and the overall incidence was 5.6% (TGC, 4.5%; usual glucose care, 6.8%). TGC was associated with decrease in dialysis (OR, 0.63; 95% CI, 0.45–0.86; Fig. 4).

TGC did not result in a significant decrease in sepsis or seizures (Fig. 4).

### Subgroup analyses

We performed three subgroup analyses by the target of TGC, by whether cardiac surgery or not, and by whether using continuous glucose monitoring for insulin adjustment. However, there were no differences between groups observed concerning those factors (Tables 3, 4 and 5).

**Table 3:** Association of tight glucose control vs usual care with outcomes among critically ill adults, stratified by tight control glucose goal

Subgroup of trials^a^	Number of trials	Event number/total number (%)		OR (95% CI)	*I*^2^ (%)
		Tight control	Usual control		
Mortality					
Very tight control	2	12/392 (3.1)	19/369 (5.1)	1.11 (0.12–10.60)	73
Moderately tight control	4	98/1582 (6.2)	94/1651 (5.7)	1.10 (0.81–1.49)	0
Overall	6	110/1974 (2.9)	113/2047 (5.5)	0.95 (0.62–1.45)	40
Any hypoglycemia (< 60 mg/dl)					
Very tight control	2	112/392 (28.6)	17/396 (4.3)	9.35 (1.49–58.83)	88
Moderately tight control	3	259/1533 (16.9)	99/1514 (6.5)	3.00 (2.07–4.34)	54
Overall	5	371/1925 (19.3)	116/1910 (6.1)	4.39 (2.39–8.06)	83
Severe hypoglycemia (< 40 mg/dl)					
Very tight control	2	24/392 (6.1)	5/396 (8.7)	5.23 (1.95–14.00)	0
Moderately tight control	3	85/1533 (5.5)	22/1514 (1.5)	3.88 (2.41–6.26)	0
Overall	5	109/1925 (18.4)	27/1910 (5.8)	4.11 (2.67–6.32)	0
Sepsis					
Very tight control	2	121/392 (30.9)	143/396 (36.1)	1.01 (0.43–2.41)	73
Moderately tight control	4	95/1582 (6.0)	132/1651 (8.0)	0.81 (0.59–1.12)	18
Overall	6	216/1947 (11.1)	275/2047 (13.4)	0.83 (0.63–1.08)	32
Dialysis					
Very tight control	1	2/349 (0.6)	6/351 (1.7)	0.33 (0.07–1.65)	NA
Moderately tight control	2	67/1184 (5.7)	97/1165 (8.3)	0.64 (0.46–0.89)	0
Overall	3	69/1533 (4.5)	103/1516 (6.8)	0.63 (0.45–0.86)	0
Seizures					
Very tight control	0	NA	NA	NA	NA
Moderately tight control	3	31/1533 (2.0)	31/1514 (2.0)	0.98 (0.59–1.63)	51
Overall	3	31/1533 (2.0)	31/1514 (2.0)	0.98 (0.59–1.63)	51

*OR* odds ratio, *CI* confidence interval, *NA* not applicable

^a^Very tight control, glucose goal < 110 mg/dl; moderately tight control, glucose goal 110–140 mg/dl

**Table 4:** Association of tight glucose control vs usual care with outcomes among critically ill adults, stratified by with cardiac surgery or without cardiac surgery

Subgroup of trials	Number of trials	Event number/total number (%)		OR (95% CI)	*I*^2^ (%)
		Tight control	Usual control		
Mortality					
Noncardiac surgery	3	57/441 (12.9)	45/394 (9.2)	1.29 (0.52–3.23)	29
Cardiac surgery	3	53/1533 (3.5)	64/1516 (4.2)	0.79 (0.50–1.26)	26
Overall	6	110/1974 (5.6)	113/2047 (5.0)	0.95 (0.62–1.45)	40
Any hypoglycemia (< 60 mg/dl)					
Noncardiac surgery	2	104/342 (30.4)	45/394 (11.4)	2.97 (2.01–4.41)	0
Cardiac surgery	3	267/1533 (17.4)	71/1516 (4.7)	5.72 (1.95–16.73)	91
Overall	5	371/1925 (19.3)	116/1910 (6.1)	4.39 (2.39–8.06)	83
Severe hypoglycemia (< 40 mg/dl)					
Noncardiac surgery	2	25/392 (6.4)	9/394 (2.3)	2.95 (1.35–6.42)	0
Cardiac surgery	3	84/1533 (5.5)	18/1516 (1.2)	4.76 (2.84–7.97)	91
Overall	5	109/1925 (18.4)	27/1910 (5.8)	4.11 (2.67–6.32)	0
Sepsis					
Noncardiac surgery	3	52/441 (11.8)	79/531 (14.9)	0.82 (0.55–1.22)	67
Cardiac surgery	3	164/1633 (10.0)	196/1516 (19.2)	0.79 (0.62–1.00)	0
Overall	6	216/1947 (11.0)	275/2047 (13.4)	0.83 (0.63–1.08)	32
Dialysis					
Noncardiac surgery	3	69/1533 (4.5)	103/1516 (6.8)	0.63 (0.45–0.86)	0
Cardiac surgery	0	NA	NA	NA	NA
Overall	3	69/1533 (4.5)	103/1516 (6.8)	0.63 (0.45–0.86)	0
Seizures					
Noncardiac surgery	1	5/349 (1.4)	10/349 (2.9)	0.49 (0.17–1.46)	NA
Cardiac surgery	2	26/1533 (1.7)	21/1165 (1.8)	1.22 (0.68–2.18)	50
Overall	3	31/1533 (2.0)	31/1514 (2.0)	0.98 (0.59–1.63)	51

*OR* odds ratio, *CI* confidence interval, *NA* not applicable

**Table 5:** Association of tight glucose control vs usual care with outcomes among critically ill adults, stratified by whether using continuous glucose monitoring (CGM)

Subgroup of trials	Number of trials	Event number/total number (%)		OR (95% CI)	*I*^2^ (%)
		Tight control	Usual control		
Mortality					
Using CGM	3	97/1553 (12.9)	86/1514 (9.2)	1.13 (0.83–1.53)	0
Not using CGM	3	13/441 (3.5)	27/533 (4.2)	0.70 (0.18–2.69)	49
Overall	6	110/1974 (5.6)	113/2047 (5.0)	0.95 (0.62–1.45)	40
Any hypoglycemia (< 60 mg/dl)					
Using CGM	3	259/1533 (30.4)	99/1514 (11.4)	3.00 (2.07–4.36)	54
Not using CGM	2	24/392 (6.1)	5/396 (4.7)	5.23 (1.95–4.00)	0
Overall	5	371/1925 (19.3)	116/1910 (6.1)	4.39 (2.39–8.06)	83
Severe hypoglycemia (< 40 mg/dl)					
Using CGM	2	85/1533 (30.4)	22/1514 (11.4)	3.88 (2.41–6.26)	0
Not using CGM	3	112/392 (28.6)	17/396 (4.7)	9.35 (1.49–58.83)	88
Overall	5	109/1925 (18.4)	27/1910 (5.8)	4.11 (2.67–6.32)	0
Sepsis					
Using CGM	3	91/1553 (12.9)	101/1514 (9.2)	0.88 (0.66–1.18)	0
Not using CGM	3	125/441 (3.5)	174/533 (4.2)	0.76 (0.18–2.69)	69
Overall	6	216/1947 (11.0)	275/2047 (13.4)	0.83 (0.63–1.08)	32
Dialysis					
Using CGM	2	67/1184 (4.5)	97/1165 (6.8)	0.64 (0.46–0.89)	0
Not using CGM	1	2/349 (4.5)	6/351 (6.8)	0.33 (0.07–1.65)	NA
Overall	3	69/1533 (4.5)	103/1516 (6.8)	0.63 (0.45–0.86)	0
Seizures					
Using CGM	3	31/1533 (2.0)	31/1514 (2.0)	0.98 (0.59–1.63)	51
Not using CGM	0	NA	NA	NA	NA
Overall	3	31/1533 (2.0)	31/1514 (2.0)	0.98 (0.59–1.63)	51

*OR* odds ratio, *CI* confidence interval, *NA* not applicable

## Discussion

### Findings and interpretations

In this meta-analysis of six RCTs of TGC vs usual care in critically ill children, we found no significant difference in risk of hospital death, sepsis, or seizures, although TGC was associated with a significant reduction in dialysis. On the other hand, we found clear evidence for the main harm of TGC: hypoglycemia increased roughly 4-fold. However, the rate of hypoglycemia varied greatly across RCTs. We performed three prespecified subgroup analyses, stratified by cardiac surgery, by continuous glucose monitoring, and by glucose goal in tight control group, to explore potential areas of bias, but subanalyses did not differ from the overall analysis. In short, our meta-analysis does not support the benefits of TGC reported in the initial trial by Vlasselaers et al. [12], yet it suggests a high risk of hypoglycemia.

### Compared with other studies

A previous meta-analysis of four RCTs examined the benefits and risks of TGC in critically ill children [16]. Similar to our findings, the meta-analysis found no significant differences in mortality but an increased risk of hypoglycemia between TGC and usual care in critically ill children. They reported, however, that TGC appeared to reduce acquired sepsis in critically ill children (OR 0.76; 95% CI 0.59–0.99). This discrepancy with our findings could be explained by the small sample size of their study. Further, we have also provided absolute as well as relative risks and a formal rating of the quality of the evidence.

We quantified a new finding, a decreased risk of dialysis with TGC. The previous meta-analysis studying the effect of TGC in critically ill children did not report the outcome of dialysis, whereas the meta-analysis of adults did not show this renoprotective effect [16, 28]. How to explain the conflicting results between our study and the other meta-analyses? One of the reasons may be the inclusion of different types of patients. The evidence for a renoprotective effect of TGC appears most pronounced in cardiac surgery patients [29]. In our study, dialysis was reported in three trials [12–14], and more than half of children in those trials underwent cardiac surgery.

### Strengths and limitations

Strengths of this review include a comprehensive search for evidence; duplicate assessment of eligibility, risk of bias, and data abstraction; and assessments of risk of bias. We included a rigorous assessment of the quality of evidence and of the credibility of subgroup analyses. Moreover, we have presented absolute and relative risks, which are crucial for making decisions regarding use of TGC in critically ill children.

Our study also has limitations. First, although there were many similarities to the methodology of the included RCTs, there was also some variability, including nutritional supplementation, target of tight glycemic control, definition of hypoglycemia, blood glucose monitoring, quality of glucose control, and duration and route used for the insulin therapy protocols. These diversities may have influenced the pathophysiology and implications of hyperglycemia. We present the findings stratified by some widely debated variables—glucose goal in the tight control group, blood glucose monitoring, and whether cardiac surgery. However, we were unable to assess the effect of other important variables for lack of adequate data.

Second, since we have pooled results from individual RCTs, our analysis is limited by any flaws in the methodology of these underlying trials. For example, all trials not using a standard care group led to variable control groups.

Third, all included studies were conducted in developed countries. Thus, our findings are applicable only to developed countries. Further research in other countries would add to the generalizability.

Fourth, the small numbers of studies and those in individual subgroup analyses limited power in our conclusions. Moreover, the limited number of included trials afforded modest ability to detect the presence of publication bias [18]. However, publication bias is unlikely as most of included RCTs had negative results.

## Conclusions

In summary, we believe the six trials included in our meta-analysis allow us to conclude about the benefits and risks of TGC critically ill children. We found that TGC was not associated with a significant reduction in hospital mortality, seizures, or sepsis, but appears to be associated with a reduction in new need for dialysis. However, TGC was associated with a markedly increased risk of hypoglycemia. These findings were consistent with recent guidelines [22, 23]. Thus, adoption of TGC in critically ill children cannot be recommended for routine use unless further high-quality and well-powered evidence shows benefit.

### Key messages

- Tight glycemic control does not appear to improve mortality in critically ill children.
- Tight glycemic control reduces a new need for dialysis in critically ill children.
- Tight glycemic control greatly increases the risk of hypoglycemia in critically ill children.

## Additional file

Additional file 1: is Supplemental Digital Content: **Table S1.** (DOCX 17 kb)

## Abbreviations

CI: Confidence interval
OR: Odds ratio
PICU: Pediatric intensive care unit
RCT: Randomized controlled trial
TGC: Tight glucose control
