# Supplementary material for: Tight glycemic control in critically ill pediatric patients: a systematic review and meta-analysis
Source: Crit Care. 2018 Mar 4;22:57. doi: 10.1186/s13054-018-1976-2 (PMC5835325; doi:10.1186/s13054-018-1976-2)
Supplement: Supplementary file 1 — is Supplemental Digital Content: Table S1. (DOCX 17 kb) [file 13054_2018_1976_MOESM1_ESM.docx]

## Appendix: Search strategy

## Ovid Medline

#1. exp insulin/ or exp blood glucose/ or strict glucose control. mp. or exp hypoglycemia/ or exp hyperglycemia/ or exp insulin infusion systems/ or intensive insulin therapy.mp.

#2. children.mp. or exp child

#3. exp intensive care units/or exp intensive care/or exp critical care/

#4 clinical trial.mp. or clinical trial.pt. or randomized.mp/ or exp randomized controlled trial

#5 1 and 2 and 3 and 4

## Cochrane CENTRAL:

(insulin or blood glucose or strict glucose control or hypoglycemia or hyperglycemia or insulin infusion systems or intensive insulin therapy) and (child or children or in infant or adolescent or preschool)

In: Title, abstract, keywords

## Ovid EMBASE

#1. exp insulin/ or exp blood glucose/ or strict glucose control. mp. or exp hypoglycemia/ or exp hyperglycemia/ or exp insulin infusion systems/ or intensive insulin therapy.mp.

#2. children.mp. or exp child

#3. exp intensive care units/or exp intensive care/or exp critical care/

#4 clinical trial.mp. or clinical trial.pt. or random:.mp. or tu.xs.

#5 1 and 2 and 3 and 4

Supplemental Digital Content- **Table S1. Glucose Goals and Mean Achieved Levels in Trials Included in the Final Meta-Analysis**

| **Author** | **Tight Control** | | **Usual Care** | |
| --- | --- | --- | --- | --- |
|  | **Glucose Goal, mg/dL** | **Glucose Achieved, Mean, mg/dL** | **Glucose Goal, mg/dL** | **Glucose Achieved, Mean, mg/dL** |
| Vlasselaers | Infants:50-80  Children:70.2-99 | Infants:86  Children:95 | 180-214 | Infants:115  Children:144 |
| Jeschke | 80-110 | NA | 140-180 | NA |
| Agus | 80-110 | 112 | Standard Care | 121 |
| Alsweiler | 72-108 | NA | 144-180 | NA |
| Marcae | 72-126 | 106 | <216 | 114 |
| Agus | 80-110 | 109 | 150-180 | 122 |

NA: Not available due to lack of data
